# Circular RNA hsa_circ_0004396 acts as a sponge of miR‐615‐5p to promote non‐small cell lung cancer progression and radioresistance through the upregulation of P21‐Activated Kinase 1

**DOI:** 10.1002/jcla.24463

**Published:** 2022-05-02

**Authors:** Dong Li, Lin Yan, Junhan Zhang, Feng Gu

**Affiliations:** ^1^ Department of Thoracic Surgery Gansu Provincial Tumor Hospital Lanzhou Gansu China; ^2^ 91589 Department of Anesthesiology Gansu Provincial Hospital Lanzhou Gansu China; ^3^ Research and Experimental Center Gansu University of Chinese Medicine Lanzhou Gansu China; ^4^ Department of Aspiration Oncology Gansu Provincial Tumor Hospital Lanzhou Gansu China

**Keywords:** miR‐615‐5p, PAK1, circRNA hsa_circ_0004396, non‐small cell lung cancer

## Abstract

**Backgrounds:**

CircRNA hsa_circ_0004396 has been confirmed to be upregulated in human non‐small cell lung cancer (NSCLC). The aim of his study was to evaluate its mechanism in the radioresistance and progression of NSCLC.

**Methods:**

Hsa_circ_0004396, miR‐615‐5p, and P21‐Activated Kinase 1 (PAK1) were measured by reverse transcription quantitative real‐time polymerase chain reaction (RT‐qPCR). The binding between miR‐615‐5p and hsa_circ_0004396 or PAK1 was predicted by circinteractome or Targetscan, as verified by dual‐luciferase reporter assay and RIP assay. Proliferation, clonogenicity capacity, cell cycle progression, apoptosis, migration, and invasion were assessed by CCK‐8, colony formation, flow cytometry, and Transwell assay. Bcl‐2, Bcl‐2 associated protein X (Bax), MMP‐2, and PAK1 protein levels were detected using western blot assay. In addition, *in vivo* function of hsa_circ_0004396 was evaluated by tumor xenograft assay.

**Results:**

Hsa_circ_0004396 and PAK1 levels were upregulated, while miR‐615‐5p was declined in NSCLC. Hsa_circ_0004396 silencing inhibited NSCLC cell malignant behavior and induced radiosensitivity. Hsa_circ_0004396 functions as a molecular sponge of miR‐615‐5p to regulate PAK1 expression. Moreover, hsa_circ_0004396 knockdown inhibited NSCLC tumor growth *in vivo*.

**Conclusion:**

Our findings demonstrated that hsa_circ_0004396 promoted NSCLC development and radioresistance through the miR‐615‐5p/PAK1 axis, which might provide a new therapeutic target for NSCLC treatment.

## Graphical abstract

CircHUWE1 boosted cell proliferation, radiosensitivity, migration and invasion and repressed apoptosis of NSCLC cells by regulating the miR‐615‐5p/PAK1 axis.

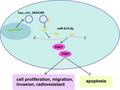

## 1 INTRODUCTION

Lung cancer is a highly lethal cancer worldwide, with non‐small cell lung cancer (NSCLC) accounting for 80%.^1^ Most advanced‐stage NSCLC sufferers are attributed to lacking an effective diagnostic method, which makes cancer hard to surgically remove and has a poor prognosis, while treatment failures are caused by radio‐ or chemoresistance.^2^, ^3^ Thus, it has important implications for further understanding the mechanisms in therapeutic resistance of NSCLC. Accordingly, the regulatory mechanism of NSCLC progression and radioresistance needs further exploration.

Circular RNA (circRNA) can be divided into noncoding circRNAs and coding circRNAs.^4^ Furthermore, circRNAs are classified as noncoding circRNAs due to their highly conserved structure, characterized by covalently closed loops lacking 5′ caps and 3′ poly tails.^5^ Although circRNAs has been discovered nearly 40 years, their biological function in many human diseases is only beginning to understand on account of the development of deep RNA sequencing (RNA‐seq) technologies and novel bioinformatic approaches.^6^, ^7^ Besides, circRNAs function as miRNA sponges, competing with pre‐mRNA splicing and serving as circRNA‐protein interactions.^8^, ^9^, ^10^, ^11^ In NSCLC, abnormal expression of circRNAs closely correlates with tumorigenesis and progression.^12^ CircMTDH.4 functioning as a miR‐630 sponge to boost growth, metastasis, and radioresistance of NSCLC cells.^13^ Also, circ_000867290 deficiency could repress cell growth and enhance the radiosensitivity of NSCLC cells via mediating the miR‐375/SPIN1.^14^ In sum, circRNAs might play vital roles in NSCLC progression and radiosensitivity. Hsa_circ_0004396jm promoted colorectal cancer cell proliferation and migration.^15^ Moreover, another research indicates that hsa_circ_0004396 is significantly upregulated in NSCLC, suggesting a potential attractive biomarker in NSCLC.^16^ However, the elaborate role and mechanism of hsa_circ_0004396 in NSCLC progression and radioresistance are unclear.

CircRNAs act as competing endogenous RNAs (ceRNAs) to sponge their target miRNAs and fulfill their regulatory function via interaction with miRNAs, regulating the miRNA‐targeted gene expression.^17^, ^18^, ^19^ Here, we first discovered by bioinformatics analysis that hsa_circ_0004396 shared binding sequence miR‐615‐5p. Moreover, some studies exhibited the suppression of miR‐615‐5p in ovarian cancer and pancreatic ductal cancer.^20^, ^21^ Also, miR‐615‐5p blocked NSCLC development through downstream mRNAs.^22^, ^23^ Interestingly, miR‐615‐5p was previously reported to be related to the radiosensitivity of human cancer.^24^ Of note, some studies suggested that P21 (RAC1) activated kinase 1 (PAK1) is an oncogenic serine/threonine kinase that is dysregulated in various cancers.^25^ Furthermore, the overexpression of PAK1 could boost chemoresistance and the development of NSCLC cells via different pathways.^26^, ^27^ In this study, we aimed to explore whether the regulatory role of hsa_circ_0004396 on NSCLC cell malignant behavior and radiosensitivity could be mediated by miR‐615‐5p/PAK1 axis.

## 2 MATERIALS AND METHODS

### 2.1 Clinical tissue samples and cell culture

This research was ratified by the Ethics Committee of Gansu Provincial Tumor Hospital. After being obtained written informed consent, NSCLC tissues (*n* = 42) and the paired adjacent normal samples were obtained between January 2016 and December 2018 at Gansu Provincial Tumor Hospital. The tissue samples were frozen in liquid nitrogen immediately and stored at −80°C for further research. None of the patients had a history of tumors or received radiochemotherapy before surgery.

A549, SPC‐A1, BEAS‐2B, and HEK293T cells were provided by BeNa, while H1299 and H460 cells were bought from ATCC. BEAS‐2B and HEK293T cells were cultured with DMEM (Gibco) contained 10% fetal bovine serum (FBS; Gibco), while A549 cells were incubated with Ham's F‐12K (Kaighn's) medium (Gibco) contained 10% fetal bovine serum and penicillin/streptomycin solution. Roswell Park Memorial Institute (RPMI) 1640 medium (Hyclone) was used to culture H1299, H460, and SPC‐A1 cells. Cells were incubated at 37°C with 5% CO2. Cell lines were authenticated by STR profiling (Microsynth) in March 2018.

### 2.2 Cell transfection

For stable knocking down of hsa_circ_0004396, short hairpin RNA against hsa_circ_0004396 was applied. Both sh‐hsa_circ_0004396 and sh‐NC (GenePharma) were constructed into the lentiviral vector (Invitrogen), followed by introduction into A549 cells and cultured in cell medium with 2 μg/ml puromycin.

Meanwhile, based on the producer’s direction of Lipofectamine 3000 (Invitrogen), miR‐615‐5p mimics/inhibitor and its control (miR‐NC/anti‐miR‐NC), empty vector (pcDNA3.1), overexpression vectors of hsa_circ_0004396 (pcDNA3.1‐hsa_circ_0004396), and PAK1 (pcDNA3.1‐PAK1) from Ribobio were transfected into 2 × 10^5^ NSCLC cells in six‐well plates.

### 2.3 RT‐qPCR

TRIzol reagent (Invitrogen) was applied to extract the total RNA from tissues and cells. For circRNA expression measurement, total RNA was purified with an RNeasy Mini kit, followed by synthesis cDNA using SuperScript™ IV First‐Strand Synthesis System (Thermo Fisher Scientific). After that, qRT‐PCR procedures were performed according to AceQ Universal SYBR qPCR Master Mix (Vazyme). Then, cDNA and reaction solutions were mixed according to protocols, and the results were recorded by Thermo Fisher QuantStudio 3 QS3 (Thermo Fisher Scientific). Mir‐X™ miRNA First‐Strand Synthesis Kit (catalog no 638315, Takara) was used for miRNA quantitation. After being normalized GAPDH and U6, data were examined through the $2^{‐\Delta \Delta C_{t}}$ method. For circRNAs stability measurement, total RNA was incubated with 5 U/μg RNase R for 20 min at 37°C or treated with actinomycin D (Act D) 2 μg/ml for 12 h to degrade the linear RNAs. The primers used in this study are shown in Table 1.

**TABLE 1:** Primer sequences used for qRT‐PCR

Primers for PCR (5′−3′)		
circHUWE1	Forward	AGGAAGTACAGGCCATGCAG
	Reverse	TGGATTGATGGCTTCTGACA
PAK1	Forward	GTGAAGGCTGTGTCTGAGACTC
	Reverse	GGAAGTGGTTCAATCACAGACCG
miR‐615‐5p	Forward	GGGGGUCCCCGGUGCUCGGAUC
	Reverse	UCCGAGCACCGGGGACCCCCUU
GAPDH	Forward	GAAGGTGAAGGTCGGAGT
	Reverse	GAAGATGGTGATGGGATTTC
U6	Forward	CTCGCTTCGGCAGCACA
	Reverse	AACGCTTCACGAATTTGCGT

### 2.4 Cell count kit‐8 (CCK‐8) assay

Briefly, NSCLC cells (2 × 10^3^ cells/well) were incubated with CCK‐8 reagent (Sangon Biotech) for 4 h after 48 h cultivation, followed by detection using Varioskan Flash 3001 (Thermo Fisher Scientific) at 450 nm.

### 2.5 Colony formation assay

The NSCLC cells (5 × 10^2^ cells/well) in the logarithmic growth phase were inoculated into the six‐well‐plates at the densities 1 × 10^3^. After incubation for 24 h, the cells were irradiated with single‐dose X‐ray (0, 2, 4, 6, and 8 Gy) based on the 6‐MV X‐ray apparatus (CX‐SN5340; ARIAN), followed by culturing with complete medium for 1 week until clone lump appeared. Cells were stained with staining liquor (G4640, Beijing Solarbio Science & Technology) for 40 min. Cloning efficiency (CE) = the clone formation quantity (at 0 Gy dose)/cell inoculated number × 100%; survival fraction (SF) = number of clones in the experimental group/number of inoculated cells × CE × 100%. A colony formation assay was conducted at least three times.

### 2.6 Flow cytometry assay

Cell apoptosis was examined by Annexin V Apoptosis Detection Kit I (Solarbio). Briefly, the residual medium was washed with phosphate buffer saline (Hyclone), and A549 and SPC‐A1 cells were re‐suspended in 100 µl binding buffer with Annexin V and Propidium iodide (PI). Ten minutes later under darkness, the samples were determined via flow cytometry.

Cell cycle progression was assessed using An FxCycle PI/RNAse Solution (F10797, Thermo Fisher Scientific). After being fixed with ethanol (75%), the cells were incubated with PI complement with DNase‐free RNase A for 1 h in darkness. Data analysis was conducted through FlowJo 10. The experiment was conducted thrice.

### 2.7 Western blot assay

Total protein was extracted using protein lysis buffer (Thermo Fisher Scientific) at 4°C. BCA Protein Assay Kit (Sangon Biotech) was used to quantitate protein concentration. The quantitative protein and 5×loading buffer were transferred to new clean centrifugal tubes in the ratio of five to one, followed by incubation in a water bath at 100°C. Ten minutes later, SDS‐PAGE (Beyotime) was implemented to separate quantitative samples (30 µg), which then were transfected onto 0.45 µm polyvinylidene fluoride (PVDF; Millipore) membrane. Non‐Fat Powdered Milk (Sangon Biotech) blocked the membrane. After being probed with primary antibodies for 2 h at room temperature, the membrane was washed three times by Tris Buffered Saline Tween (TBST, Sangon Biotech). Then, the secondary HRP‐conjugated antibody was used to incubate the membrane for 40 min to combine the matched primary antibody. TBST was used to wash off residual solution four times. Finally, special proteins were visualized by SuperSignal Chemiluminescent Substrates (Thermo Fisher Scientific). The antibodies were purchased from Abcam, including matrix metalloproteinase‐2 (MMP‐2, catalog no ab92536), Bax (catalog no ab32503), B‐cell lymphoma‐2 (Bcl‐2, catalog no ab196495), PAK1 (catalog no ab154284) and GAPDH (catalog no ab9485), and secondary antibody (catalog no ab205718, 1:20,000).

### 2.8 Transwell assay

For migration assay, NSCLC cells (5 × 10^4^ cells/well) were seeded in the upper chambers with 200 µl serum‐free cell medium, and 500 µl medium containing 10% FBS was added into the lower chamber. Then, the cells were incubated at 37°C for 24 h. Subsequently, crystal violet (MedChemExpress) was used to stain the cells for 30 min. The stained cells were observed under an inverted microscope (CarlZeiss). To detect cell invasion, 1 × 10^5^ cells were introduced into the upper counterpart pre‐covered with Matrigel Matrix, and the subsequent steps were the same as cell migration assay.

### 2.9 Dual‐luciferase reporter assay

The relationship between miR‐615‐5p and hsa_circ_0004396 or PAK1 was predicted by Starbase. The fragments of wild‐type (hsa_circ_0004396‐WT) and mutant (hsa_circ_0004396‐MUT) hsa_circ_0004396 containing presumptive sites for miR‐615‐5p were generated by Ribobio. Then, the above sequences were constructed into the pmirGLO vector (Promega). Likewise, PAK1‐WT and PAK1‐MUT vectors were also constructed according to the above description. For dual‐luciferase reporter assay, particular vectors were co‐transfected into HEK293T cells with miR‐615‐5p mimic or miR‐NC. Assessment of luciferase activities of renilla and firefly was conducted using Dual‐Luciferase Assay Kit (Promega), and renilla activity was regarded as an internal control for standardizing firefly activity.

### 2.10 RNA immunoprecipitation (RIP) assay

Non‐small cell lung cancer cells were lysed by RIP lysis buffer (Beyotime), followed by a mixture with magnetic beads and anti‐Ago2 or anti‐IgG (Millipore). After digestion, immunoprecipitated RNA was analyzed.

### 2.11 *In vivo* tumor formation assay

All animal experimental procedures were approved by the Institutional Animal Care and Use Committee of Gansu Provincial Tumor Hospital. For the establishing of the xenograft model, A549 cells (1 × 10^7^) with sh‐NC or sh‐hsa_circ_0004396 were subcutaneously injected into the nude mice (4 weeks old, 5 mice/group). At the same time, mice were checked daily with normal activities such as eating, drinking, eliminating, and ambulating. Tumor size was measured every 5 days, followed by calculation following the formula that length × width^2^/2. After injection for 30 days, the mice were sacrificed and the tumor weight was measured. Immunohistochemical staining assay was carried out as described previously,^28^ and tissue sections from the obtained tumors were incubated with antibodies specific for Ki67 (0.5 μg/ml, ab15580; Abcam) and (1:100, ab223849; Abcam).

### 2.12 Statistical analysis

Data were exhibited as mean ± standard deviation (SD). Significance of the variance was analyzed using Student’s *t* test or one‐way analysis of variance (ANOVA). Significant differences were defined as *p* < 0.05.

## 3 RESULTS

### 3.1 Hsa_circ_0004396 was upregulated in NSCLC

**FIGURE 1:**
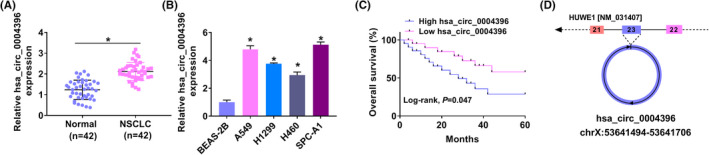
Expression of hsa_circ_0004396 was upregulated in NSCLC. (A and B) hsa_circ_0004396 was detected by qRT‐PCR in NSCLC tissues (A) and cell lines (B). (C) Kaplan–Meier curve analysis of overall survival in hsa_circ_0004396 expressions of NSCLC patients. (D) Schematic illustration showing the circularization of HUWE1 exon 21‐23 forming hsa_circ_0004396. *p < 0.05

QRT‐PCR showed a significant increase of hsa_circ_0004396 in NSCLC (Figure 1A,B). Moreover, hsa_circ_0004396 low expression (divided according to the median) tends to have much better survival rates within a 5‐year statistical period than the hsa_circ_0004396 high expression group (Figure 1C). Meanwhile, our result exhibited that hsa_circ_0004396 was originated from exons 21, 22, and 23 of the HUWE1 gene (Figure 1D). CircRNAs are very stable due to their unique covalent loop structure. As expected, the RNase R treatment resulted in the obvious decrease in HUWE1 mRNA, whereas the hsa_circ_0004396 was unaffected in NSCLC cells (Figure S1A,B). The transcription inhibitor actinomycin D (Act D) allowed an assessment of RNA decay over time. As shown in Figure S1C,D, hsa_circ_0004396 showed more outstanding resistance than HUWE1 mRNA. The above results further confirmed that hsa_circ_0004396 was a stable circRNA. Furthermore, Table 2 displayed patients’ clinicopathologic parameters. The expression of hsa_circ_0004396 correlates with the TNM stage and Lymph node metastasis, implying the potential role of hsa_circ_0004396 in the forming and developing of NSCLC.

**TABLE 2:** Clinicopathologic parameters and circHUWE1 expression in patients with NSCLC

Parameters	Case	circHUWE1 expression		^a^ *P* value
		High (*n* = 21)	Low (*n* = 21)	
Age				
<65	20	9	11	0.360
≥65	22	12	10	
Gender				
Female	16	9	7	0.412
Male	26	12	14	
Smoking				
No	19	11	8	0.504
Yes	23	10	13	
TNM stage				
I + II	27	11	16	0.033*
III	15	10	5	
Lymph node metastasis				
No	13	4	9	0.025*
Yes	29	17	12	

Abbreviations: circHUWE1, hsa_circ_0004396; NSCLC, non‐small cell lung cancer; TNM, tumor node metastasis.

^a^ Chi‐square test.

* *p* < 0.05.

### 3.2 Hsa_circ_0004396 deficiency suppressed NSCLC cell malignant behavior and increased radiosensitivity

**FIGURE 2:**
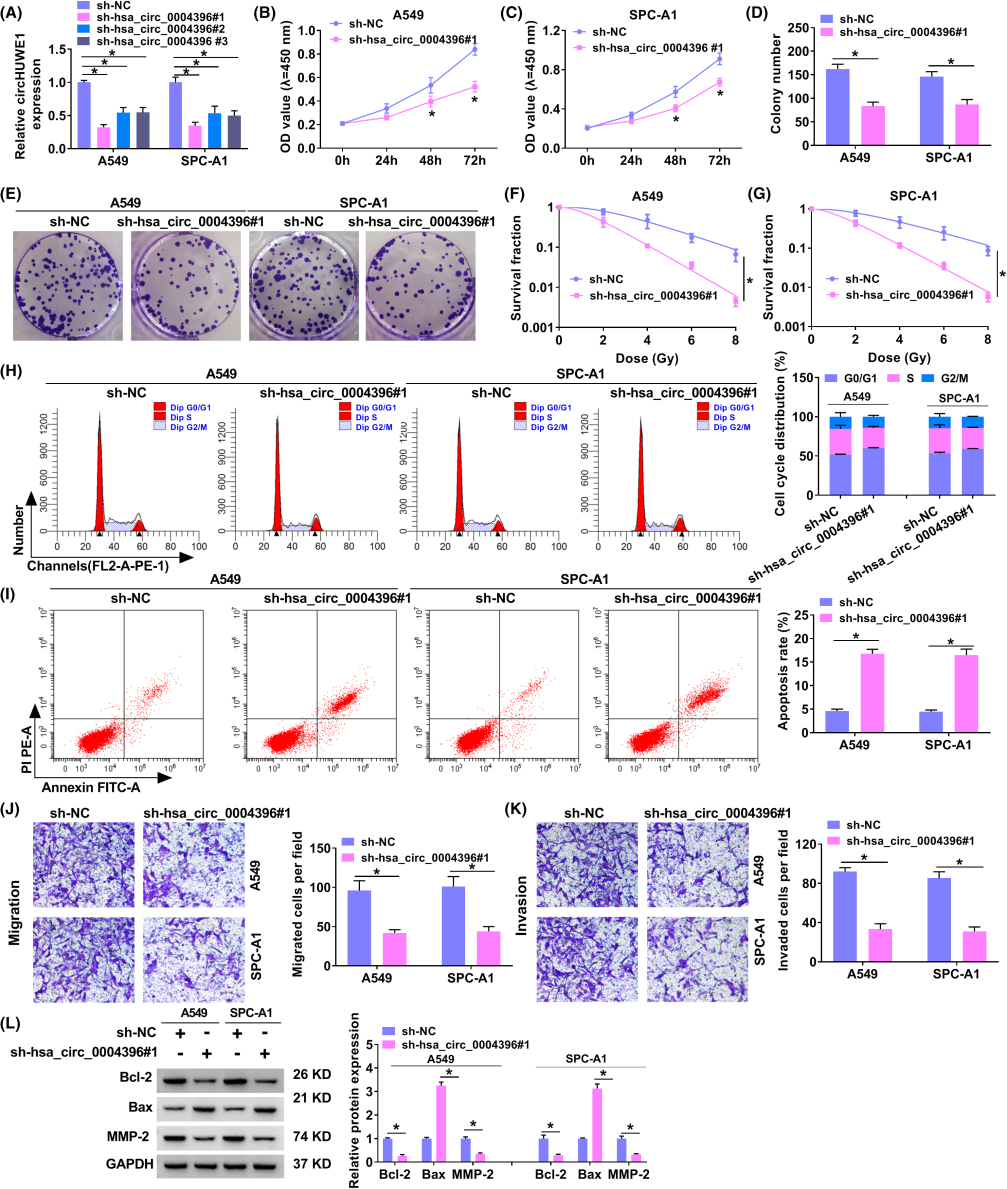
hsa_circ_0004396 silencing suppressed NSCLC cell malignant behavior and increased radiosensitivity. (A) Hsa_circ_0004396 was evaluated using qRT‐PCR in A549 and SPC‐A1 cells transfected with sh‐hsa_circ_0004396#1, sh‐hsa_circ_0004396#2, sh‐hsa_circ_0004396#3, and sh‐NC. (B–K) tumor cells were transfected with sh‐hsa_circ_0004396#1. (B and C) CCK‐8 assay was performed to detect cell proliferation of A549 (B) and SPC‐A1 (C) cells after transfection. (D and E) Colony formation assay was conducted to detect the cell vitality of A549 and SPC‐A1 cells. (F and G) Tumor cells were radiated at different doses ranging from 0–8 Gy, followed by analysis using colony formation assay. (H and I) Cell cycle progression (H) and cell apoptosis (I) were examined by flow cytometry *in vitro*. (J and K) Transwell assay was carried out to determine cell migration (J) and invasion (K) in NSCLC cells. (L) The protein levels of Bcl‐2, Bax, and MMP‐2 were detected by Western blot. *p < 0.05

Small interfering RNA against hsa_circ_0004396 (sh‐hsa_circ_0004396#1, sh‐hsa_circ_0004396#2, and sh‐hsa_circ_0004396#3) and sh‐NC were transfected into NCCLC cells, followed by verification knockdown efficiency using qRT‐PCR (Figure 2A). Due to significant knockdown efficiency, sh‐hsa_circ_0004396#1 was chosen for further experiments. Cell viability was significantly suppressed by sh‐hsa_circ_0004396#1 in NSCLC cells (Figure 2B–E). Apart from that, we also detected whether hsa_circ_0004396 is associated with the radiotherapy effect. The results showed that the hsa_circ_0004396 knockdown group has a higher survival fraction in a dose‐dependent manner (Figure 2F,G). Hsa_circ_0004396 downregulation induced G0/G1 arrest and apoptosis in NSCLC cells (Figure 2H,I). Furthermore, migration and invasion were both constrained via hsa_circ_0004396 knockdown (Figure 2J,K). Then, protein levels of Bcl‐2 (a pro‐apoptosis factor), Bax (an anti‐apoptosis factor), and MMP‐2 (a pro‐migration and invasion factor) were assessed. Data indicated that the decreased protein level of Bcl‐2 and the increased protein levels of Bax and MMP‐2 were viewed due to hsa_circ_0004396 downregulation (Figure 2L). Taken together, downregulation of hsa_circ_0004396 suppressed cell malignant behavior and increased radiosensitivity in NSCLC cells *in vitro*.

### 3.3 MiR‐615‐5p was a target of hsa_circ_0004396

**FIGURE 3:**
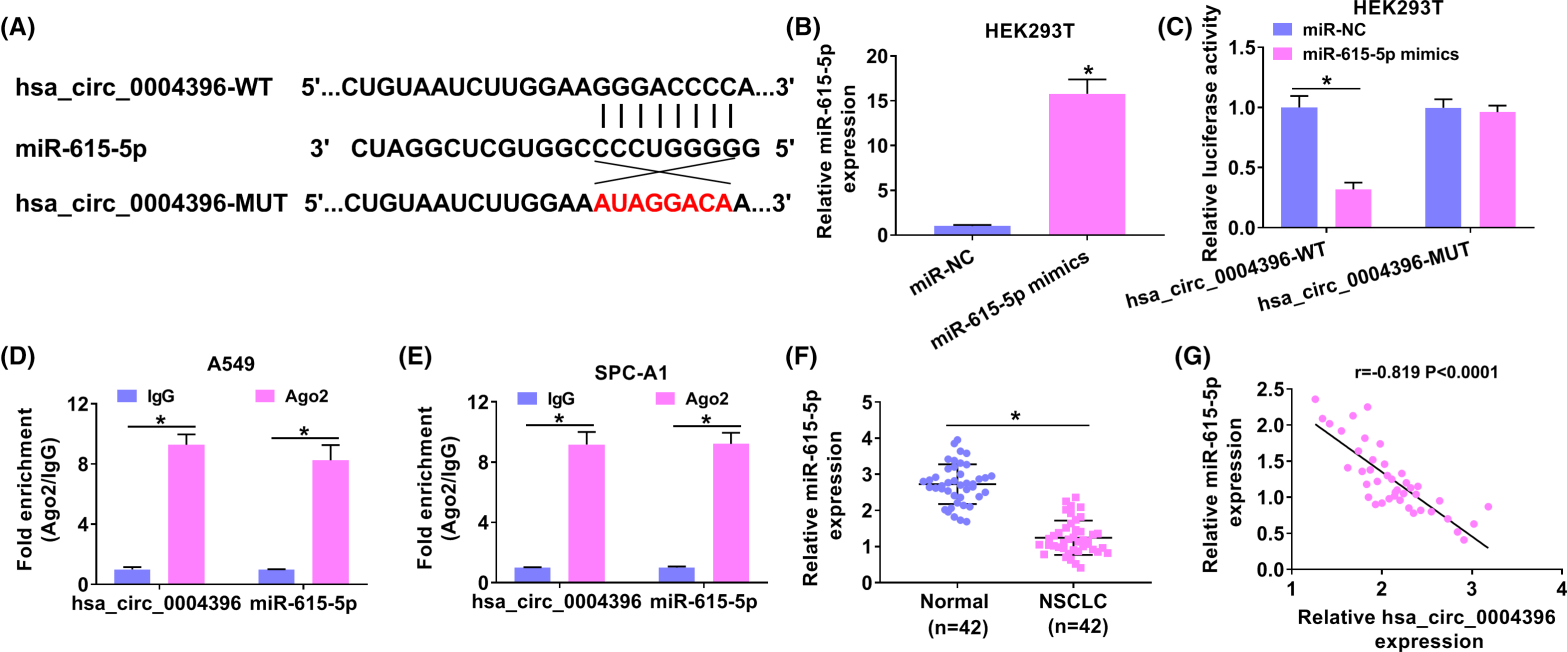
MiR‐615‐5p was a target of hsa_circ_0004396. (A) The binding sites of hsa_circ_0004396‐WT or hsa_circ_0004396‐MUT in miR‐615‐5p were predicted by the Circinteractome database. (B and C) A Dual‐luciferase reporter assay was conducted to verify the interaction between miR‐615‐5p and hsa_circ_0004396. (D and E) Anti‐Ago2 RIP assay was conducted in tumor cells. (F) qRT‐PCR analysis of miR‐615‐5p in NSCLC tissues and normal tissues. (G) Expression correlation between hsa_circ_0004396 and miR‐615‐5p in NSCLC tissues was analyzed by Spearman's rank analysis. *p < 0.05

Circinteractome predicted the combinative sequence between hsa_circ_0004396 and miR‐615‐5p, and binding sites are shown in Figure 3A. Luciferase activity in HEK293T cells was significantly decreased in the hsa_circ_0004396‐WT group, while the luciferase activity was changed little in the hsa_circ_0004396‐MUT group, suggesting hsa_circ_0004396 sponged miR‐615‐5p (Figure 3B,C). Then, enrichments of hsa_circ_0004396 and miR‐615‐5p were greatly increased when incubated with Anti‐Argonaute‐2 antibody (anti‐Ago2) using Ago2 RIP assay (Figure 3D,E). Apart from that, the expression level of miR‐615‐5p was downregulated in NSCLC tissue samples (Figure 3F) and negatively correlated with the expression of hsa_circ_0004396 (Figure 3G).

Moreover, a notably decreased miR‐615‐5p was found in NSCLC cells (Figure S2A). In addition, the overexpression efficiency of hsa_circ_0004396 was detected and presented in Figure S2B. After that, upregulation of hsa_circ_0004396 decreased the expression of miR‐615‐5p (Figure S2C). Inversely, the hsa_circ_0004396 knockdown improved miR‐615‐5p level (Figure S2D). All in all, hsa_circ_0004396 interacted with miR‐615‐5p.

### 3.4 MiR‐615‐5p suppressed cell malignant behavior and increased radiosensitivity

**FIGURE 4:**
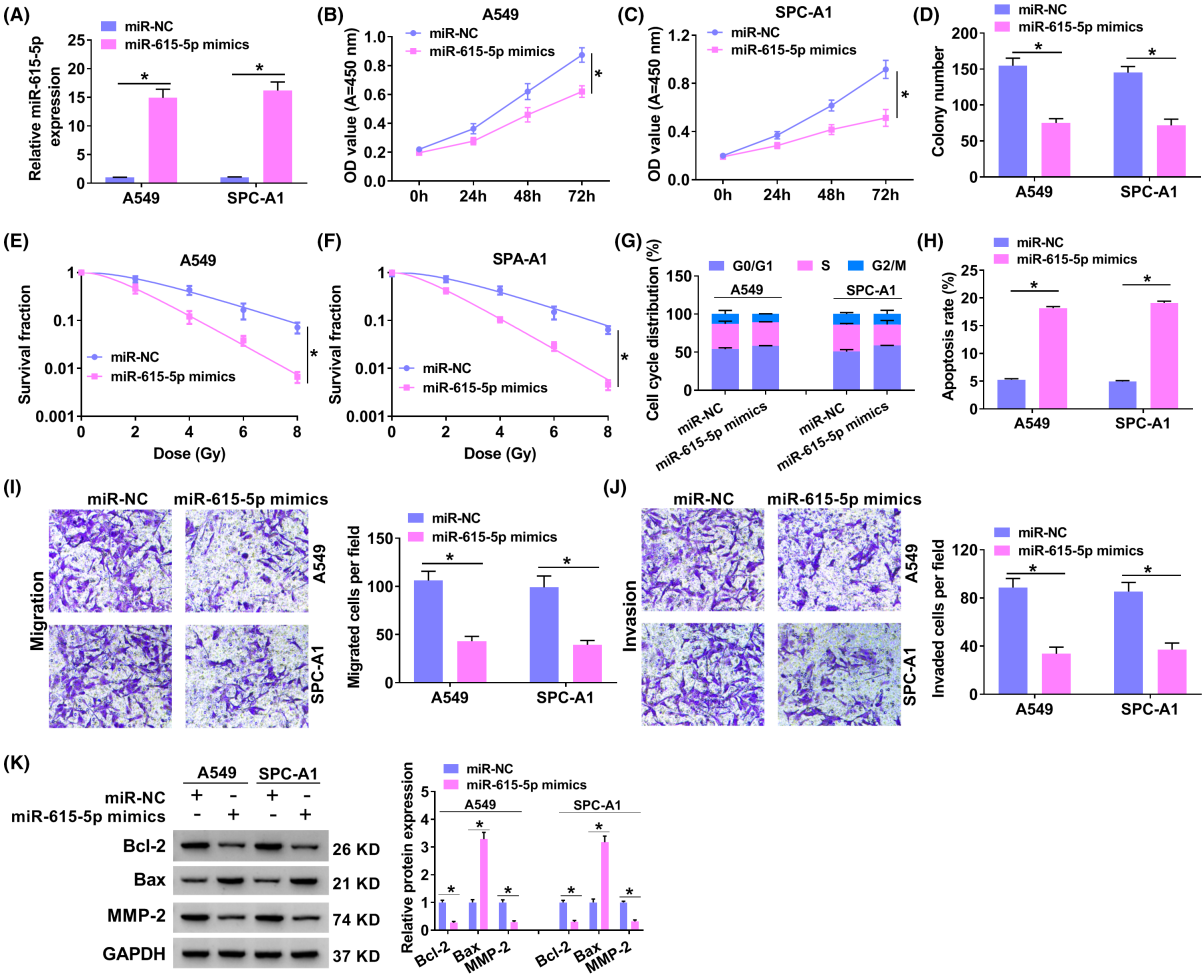
MiR‐615‐5p suppressed NSCLC cell malignant behavior and increased radiosensitivity. (A‐K) A549 and SPC‐A1 cells were transfected with miR‐NC or miR‐615‐5p mimics. (A) miR‐615‐5p was evaluated using qRT‐PCR. (B and C) CCK‐8 assay was used to detect cell proliferation of A549 (B) and SPC‐A1 (C) cells after transfection. (D) Colony formation assay was conducted to detect the cell vitality after transfection. (E and F) Colony formation assay was conducted to detect the cell survival fraction. (G and H) Cell cycle progression (G) and cell apoptosis (H) was examined by flow cytometry. (I and J) Transwell assay was carried out to determine cell migration (I) and invasion (J) in NSCLC cells. (K) The levels of Bcl‐2, Bax, and MMP‐2 protein were detected by Western blot. *p < 0.05

As shown in Figure 4A, the introduction of miR‐615‐5p mimics was able to enhance miR‐615‐5p in tumor cells. Cell viability was repressed by miR‐615‐5p overexpression in NSCLC cells (Figure 4B–D). Moreover, the miR‐615‐5p overexpression also enhanced radiosensitivity in A549 and SPC‐A1 cells (Figure 4E,F). Besides, flow cytometry revealed that miR‐615‐5p mimics induced G0/G1 arrest (Figure 4G) and promoted apoptosis in A549 and SPC‐A1 cells (Figure 4H). Migration and invasion abilities were hindered caused by miR‐615‐5p upregulation in tumor cells (Figure 4I,J). Additionally, changes in Bcl‐2, Bax, and MMP‐2 protein levels also further these effects (Figure 4K). Overall, miR‐615‐5p hindered NSCLC cell development.

### 3.5 PAK1 was directly targeted by miR‐615‐5p

Considering the binding between miR‐615‐5p and PAK1 (Figure S3A), their interaction in HEK293T cells was verified. As exhibited in Figure S3B, the luciferase activity was significantly blocked in the wild‐type group, while there was no significant difference in the mutant group. Also, miR‐615‐5p and PAK1 were enriched in the Ago2 group vs. the IgG group using RIP assay (Figure S3C,D). Besides, PAK1 expression was remarkably increased in NSCLC cells (Figure S3E,F). Meanwhile, miR‐615‐5p was significantly decreased by a miR‐615‐5p inhibitor (Figure S3G). PAK1 was enhanced via miR‐615‐5p mimic but reduced via miR‐615‐5p downregulation in tumor cells (Figure S3H–K). Furthermore, compared with the normal tissues, the levels of PAK1 protein (Figure S3L) and mRNA (Figure S3M) were distinctly increased in NSCLC tissues (Figure S3J). Interestingly, in NSCLC tissues, PAK1 level was negatively correlated with miR‐615‐5p and positively associated with hsa_circ_0004396 level (Figure S3N,O), implying the relationship of hsa_circ_0004396, miR‐615‐5p, and PAK1 in NSCLC. Overall, these data indicated that PAK1 was targeted by miR‐615‐5p.

### 3.6 Hsa_circ_0004396 regulated cell malignant behavior and radiosensitivity by miR‐615‐5p/PAK1 *in vitro*

**FIGURE 5:**
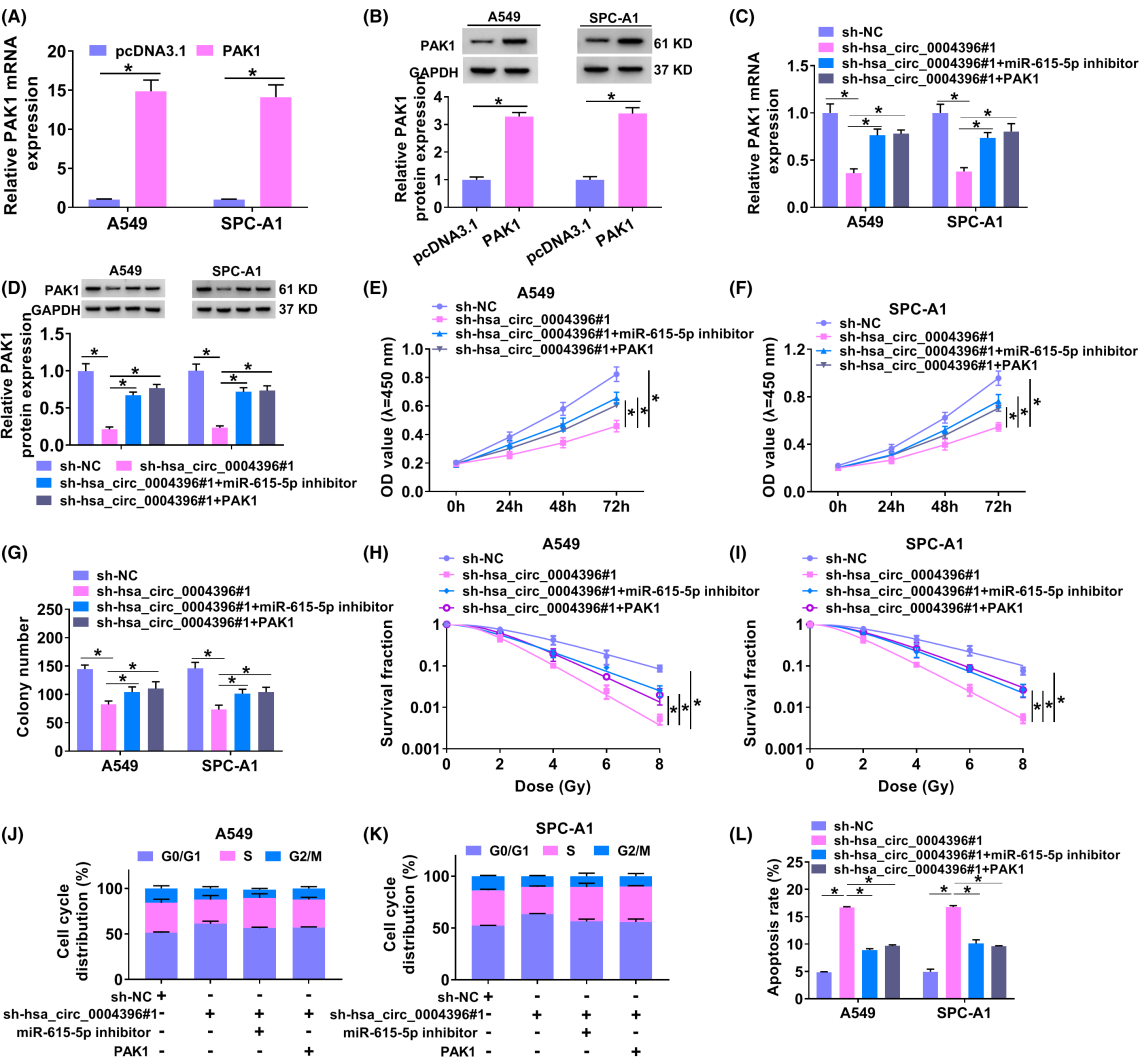
Hsa_circ_0004396 regulated NSCLC cell malignant behavior and radiosensitivity by miR‐615‐5p/PAK1 *in vitro*. (A and B) PAK1 in NSCLC cells transfected with pcDNA3.1 or PAK1 were detected by qRT‐PCR and Western blot. (C‐L) A549 and SPC‐A1 cells were transfected with sh‐NC, sh‐hsa_circ_0004396#1, sh‐hsa_circ_0004396#1 + miR‐615‐5p inhibitor, or sh‐hsa_circ_0004396#1 + PAK1 respectively. (C and D) PAK1 in NSCLC cell lines was examined via qRT‐PCR assay and Western blot. (E and F) CCK‐8 was used to detect cell proliferation of A549 (E) and SPC‐A1 cells (F) after transfection. (G) Colony formation assay was conducted to examine the cell viability after transfection. (H and I) Colony formation assay detects the cell survival fraction. (J–L) cell cycle progression and apoptosis in tumor cells were assessed using flow cytometry. *p < 0.05

**FIGURE 6:**
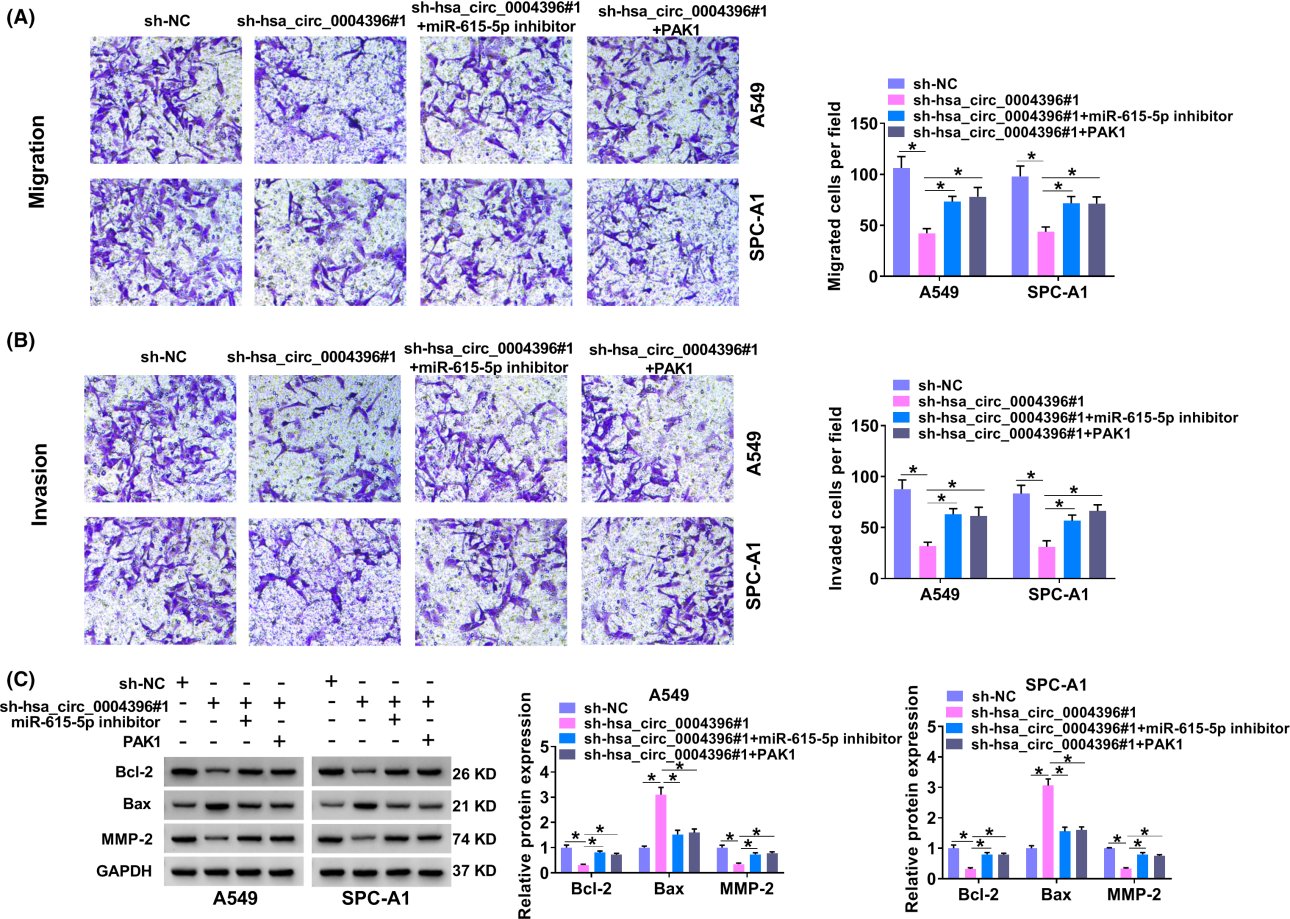
Hsa_circ_0004396 regulated migration and invasion by miR‐615‐5p/PAK1 *in vitro*. (A–C) A549 and SPC‐A1 cells were transfected with sh‐NC, sh‐hsa_circ_0004396#1, sh‐hsa_circ_0004396#1 + miR‐615‐5p inhibitor, or sh‐hsa_circ_0004396#1 + PAK1. (A and B) Transwell assay was aimed to identify cell migration (A) and invasion (B) in transfected A549 and SPC‐A1 cells. (C) The levels of Bcl‐2, Bax, and MMP‐2 protein in transfected A549 and SPC‐A1 cells were analyzed by Western blot assay. *p < 0.05

As hsa_circ_0004396 targets miR‐615‐5p and PAK1 is a target of miR‐615‐5p, we further confirmed whether hsa_circ_0004396 exerts its role via miR‐615‐5p/PAK1 axis. Also, the introduction of pcDNA3.1‐PAK1 might improve the PAK1 level in NSCLC cells (Figure 5A,B). Subsequently, A549 and SPC‐A1 cells were transfected with sh‐NC, sh‐hsa_circ_0004396#1, sh‐hsa_circ_0004396#1 + miR‐615‐5p inhibitor, or sh‐hsa_circ_0004396#1 + PAK1 respectively. As shown in Figure 5C,D, PAK1 was decreased by hsa_circ_0004396 knockdown, which was reversed using miR‐615‐5p downregulation or PAK1 overexpression. Moreover, the inhibiting action of sh‐hsa_circ_0004396#1 on cell viability was also partially rescued by miR‐615‐5p downregulation or PAK1 overexpression (Figure 5E–G). Similarly, hsa_circ_0004396 downregulation induced an increase in sensitivity to radiotherapy in A549, and SPC‐A1 cells were inverted via miR‐615‐5p inhibitor or PAK1 (Figure 5H,I). Additionally, miR‐615‐5p knockdown or PAK1 upregulation also reversed hsa_circ_0004396 knockdown‐triggered cell apoptosis and cycle progression promotion (Figure 5K–L), accompanied with lower Bcl‐2 and higher Bax (Figure 6C). Besides, the suppressive role of hsa_circ_0004396 knockdown on cell migration and invasion in tumor cells was partially restored using miR‐615‐5p reduction or PAK1 elevation (Figure 6A,B), as evidenced by decreased MMP‐2 level (Figure 6C). Taken together, hsa_circ_0004396 regulated cell malignant behavior and radiosensitivity by miR‐615‐5p/PAK1 *in vitro*.

### 3.7 Knockdown of hsa_circ_0004396 inhibited tumor growth in NSCLC *in vivo*

**FIGURE 7:**
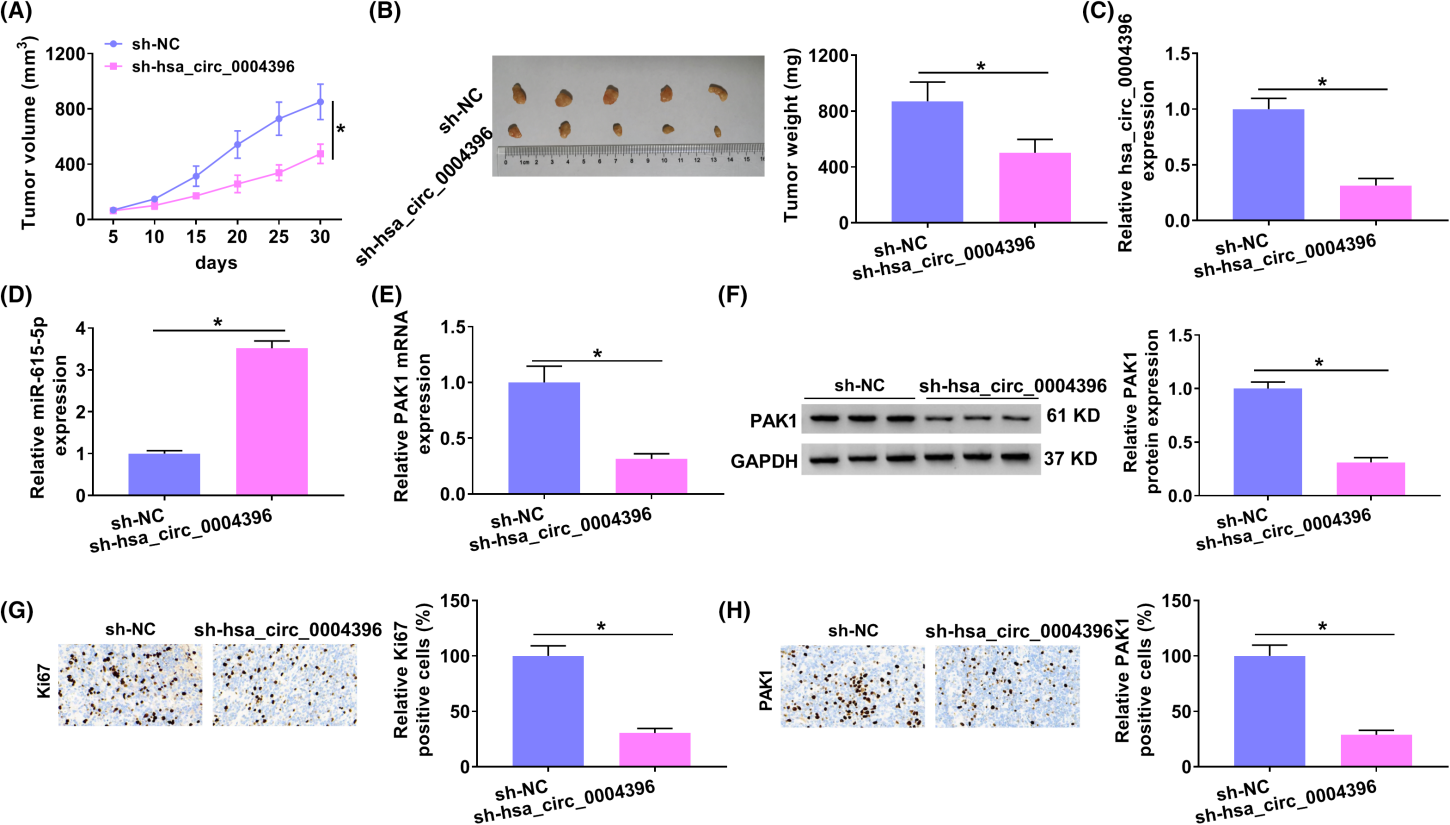
Knockdown of hsa_circ_0004396 inhibited tumor growth in NSCLC *in vivo*. (A and B) The tumor volume and weight were measured. (C and D) hsa_circ_0004396 and miR‐615‐5p were detected by qRT‐PCR. (E and F) The mRNA and protein levels of PAK1 were detected by qRT‐PCR and Western blot. (G and H) The expression levels of Ki67 and PAK1 were examined by Immunohistochemical staining. *p < 0.05

To further investigate the function of hsa_circ_0004396 in NSCLC *in vivo*, xenograft formation assay was performed in nude mice. Tumor growth was significantly suppressed by hsa_circ_0004396 knockdown (Figure 7A,B). Subsequently, qRT‐PCR results exhibited that hsa_circ_0004396 expression was reduced, while miR‐615‐5p expression was increased in the sh‐hsa_circ_0004396 group compared with the sh‐NC group (Figure 7C,D). PAK1 was downregulated in NSCLC transplanted tumor tissues *in vivo* (Figure 7E,F). In addition, Ki67 (a proliferation marker) and PAK1 expression were inhibited by hsa_circ_0004396 downregulation using immunohistochemical staining (Figure 7G,H). Our data revealed that hsa_circ_0004396 promoted NSCLC development *in vivo* through regulating miR‐615‐5p and PAK1.

## 4 DISCUSSION

Non‐small cell lung cancer occupies approximately 80% of lung cancer, is the leading cause of cancer death.^29^ circRNAs and miRNAs exhibited critical roles in the carcinogenesis of malignant tumors from some studies.^12^, ^19^ In this study, we found that hsa_circ_0004396, a typical circRNA, was upregulated in NSCLC cells and tissues, consistent with the former work.^16^ Moreover, hsa_circ_0004396 high expression was positively related to NSCLC sufferers’ poor prognosis. Moreover, knockdown of hsa_circ_0004396 inhibited NSCLC cell malignant behavior and induced radiosensitivity. This suggested that hsa_circ_0004396 may act as an oncogene in the progression of NSCLC. Consistently, hsa_circ_0004396 silencing repressed NSCLC cell growth *in vivo*. In consequence, hsa_circ_0004396 may serve as a potential attractive biomarker for diagnosis and prognosis.

As we have mentioned, circRNA can bind with its target miRNAs via sponging miRNAs, regulating the miRNA‐targeted gene expression. The current work proved that hsa_circ_0004396 targeted miR‐615‐5p. Past research demonstrated that miR‐615‐5p performed a key role in carcinogenesis and the development of malignant tumors.^30^, ^31^, ^32^ Furthermore, miR‐615‐5p was previously validated to suppress cell growth and metastasis of NSCLC cells.^22^, ^23^ Meanwhile, it has been confirmed that miR‐615‐5p can take part in the regulation of radiosensitivity in prostate cancer cells.^24^ In agreement with these reports, miR‐615‐5p could inhibit NSCLC cell progression and radioresistance in this article.

Moreover, we further confirmed that miR‐615‐5p targeted the PAK1. PAK1, an oncogene, encodes a family member of serine/threonine p21‐activating kinases. Numerous studies indicated that PAK1 participated in cancers progression and was related to poor prognosis in gastric cancer^33^, ^34^ and thyroid cancer.^35^, ^36^ Additionally, PAK1 correlates with the radiosensitivity of cancers. β‐elemene was able to enhance the radiosensitivity of gastric cancer cells by inhibiting PAK1 activation.^37^ PAK1 tyrosine phosphorylation is necessary to induce epithelial–mesenchymal transition and radioresistance in lung cancer cells.^38^ Our research demonstrated that PAK1 was targeted by miR‐615‐5p in NSCLC. Also, miR‐615‐5p downregulation or PAK1 upregulation partly reversed hsa_circ_0004396 knockdown‐mediated NSCLC cell progression and radioresistance repression in this work, and further verifying hsa_circ_0004396 could regulate the progression and radioresistance in NSCLC by targeting the miR‐615‐5p/PAK1 axis. However, we failed to detect the downstream signal transduction mechanisms and we are interested in molecular mechanisms by which the hsa_circ_0004396 /miR‐615‐5p/PAK1 axis exert their function in NSCLC.

**FIGURE 8:**
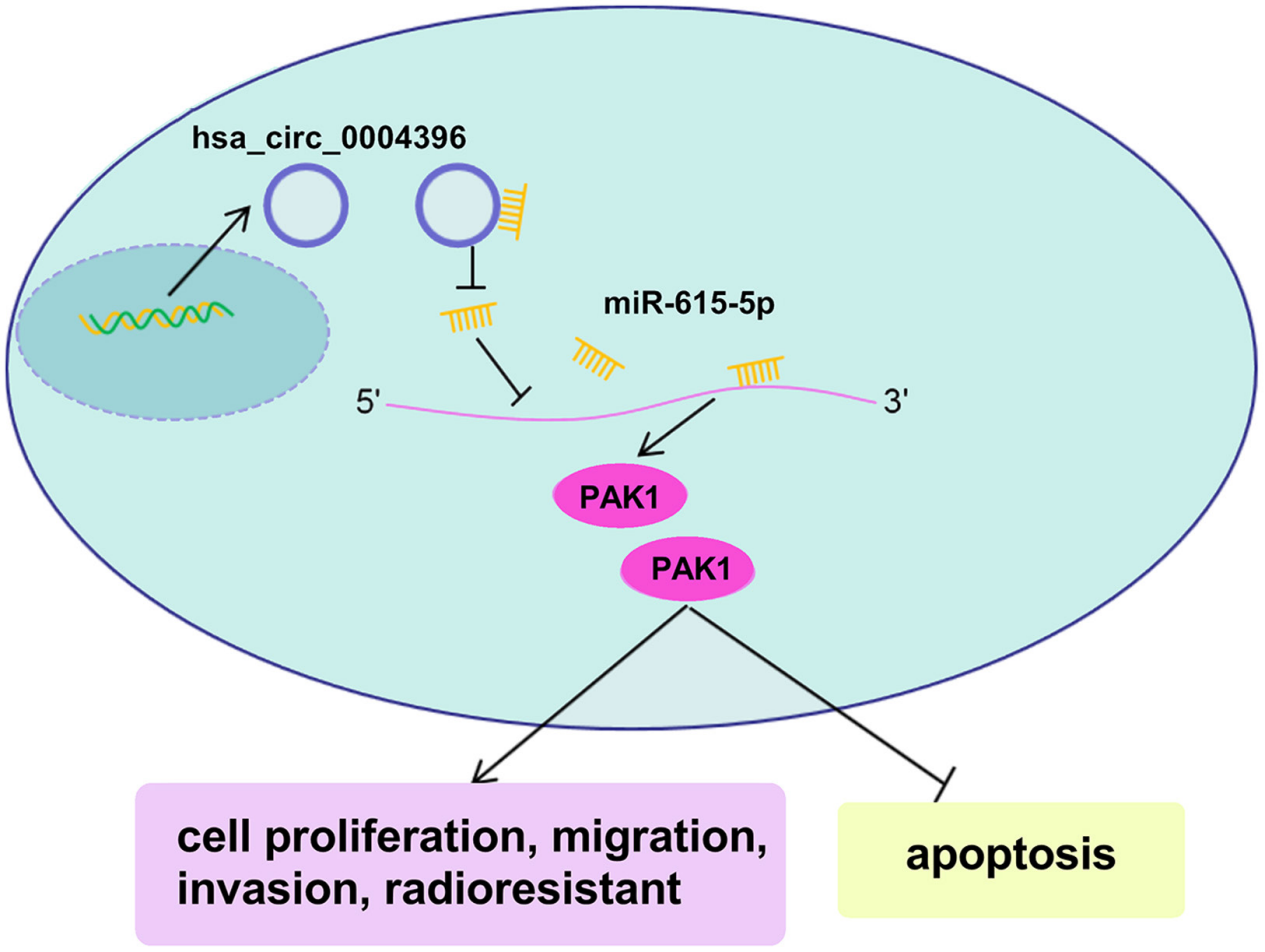
Hsa_circ_0004396 boosted NSCLC cell malignant behavior and radiosensitivity, by regulating the miR‐615‐5p/PAK1 axis

In summary, hsa_circ_0004396 sponged miR‐615‐5p to regulate PAK1, which ultimately inhibits NSCLC cell malignant behavior and radiosensitivity (Figure 8). The functions of hsa_circ_0004396 in NSCLC cells were partially rescued by miR‐615‐5p inhibition or PAK1 overexpression. Furthermore, hsa_circ_0004396 promoted NSCLC progression via regulating miR‐615‐5p and PAK1 *in vivo*. The hsa_circ_0004396/miR‐615‐5p/PAK1 axis might provide a novel direction for further study in NSCLC.

## CONFLICT OF INTEREST

The authors have no interests to disclose.

## Supporting information

Fig S1

Fig S2

Fig S3

## Data Availability

The datasets used and/or analyzed during the current study are available from the corresponding author on reasonable request.
