# Supplementary figures and images for: Circular RNA hsa_circ_0004396 acts as a sponge of miR‐615‐5p to promote non‐small cell lung cancer progression and radioresistance through the upregulation of P21‐Activated Kinase 1
Source: J Clin Lab Anal. 2022 May 2;36(6):e24463. doi: 10.1002/jcla.24463 (PMC9169218; doi:10.1002/jcla.24463)

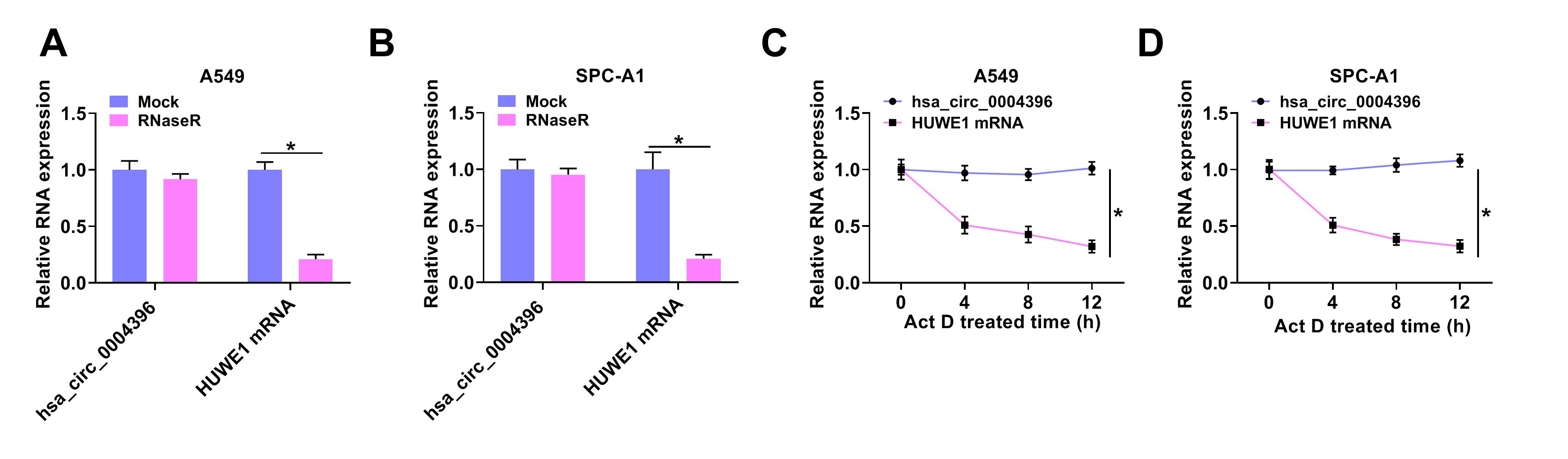

Supplement: Supplementary file 1 — Fig S1 [file JCLA-36-e24463-s001.tif]

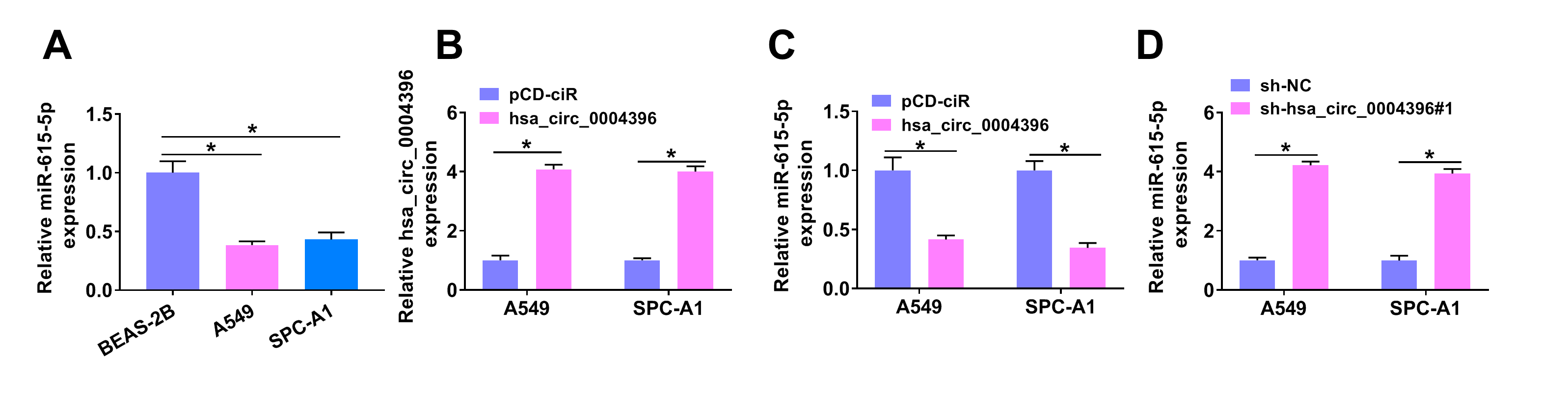

Supplement: Supplementary file 2 — Fig S2 [file JCLA-36-e24463-s003.tif]

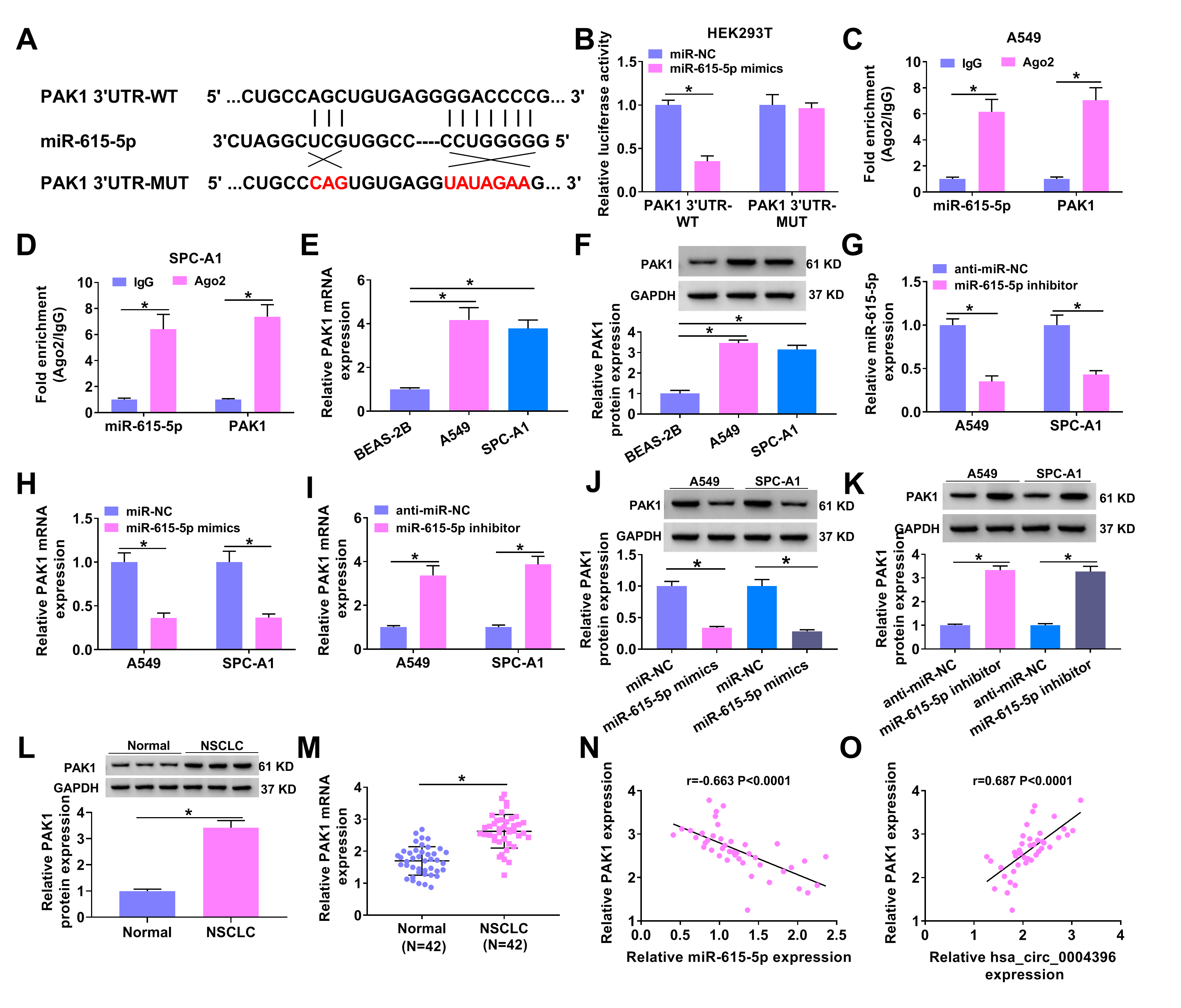

Supplement: Supplementary file 3 — Fig S3 [file JCLA-36-e24463-s002.tif]
